# The relationship between levothyroxine dosage and free thyroxine levels in hypothyroid patients: a large retrospective study

**DOI:** 10.1530/ETJ-24-0388

**Published:** 2025-07-17

**Authors:** Toshihiko Kasahara

**Affiliations:** Kasahara Clinic, Chuo-ku, Osaka, Japan

**Keywords:** levothyroxine (LT4), free thyroxine (FT4), FT3/FT4 ratio, hypothyroidism, immunoassay

## Abstract

**Objective:**

This study examined the relationship between levothyroxine dosage and free thyroxine levels in hypothyroid patients. The aim was to ascertain whether elevated free thyroxine in treated patients suggests overmedication or is essential for maintaining appropriate free triiodothyronine levels, guiding improved monitoring practices during therapy.

**Methods:**

A retrospective analysis was conducted on 3,020 free thyroxine measurements from 1,409 patients between July 2021 and March 2024. Patients with thyrotropin receptor antibodies or treated with antithyroid drugs such as thiamazole, propylthiouracil, and potassium iodide were excluded. Measurements were performed using the Elecsys FT4 III immunoassay, and statistical comparisons were made between levothyroxine-treated and untreated groups.

**Results:**

Levothyroxine-treated patients showed significantly higher median free thyroxine levels (17.9 pmol/L, interquartile range (IQR): 15.6–20.1) than untreated patients (16.2 pmol/L, IQR: 14.5–17.9, *P* < 0.0001). In addition, the free triiodothyronine/free thyroxine ratio was significantly lower in levothyroxine-treated patients (0.24, IQR: 0.20–0.29) than in untreated patients (0.28, IQR: 0.25–0.32, *P* < 0.0001). Free thyroxine levels increased with levothyroxine dosage, whereas the free triiodothyronine/free thyroxine ratio decreased. Although thyroid-stimulating hormone levels did not differ significantly between the groups, higher levothyroxine doses were associated with mild suppression.

**Conclusion:**

The findings emphasize the importance of higher free thyroxine levels for maintaining adequate free triiodothyronine in levothyroxine-treated patients, underscoring the need to monitor free thyroxine, free triiodothyronine, and their ratio during therapy to optimize treatment outcomes. In addition, clinicians should recognize that higher levothyroxine doses may elevate free thyroxine levels beyond the reference range.

## Introduction

The thyroid gland primarily produces thyroxine (T4) and triiodothyronine (T3), with T4 being the predominant hormone. These hormones regulate metabolism, growth, and development (1). Accurate management of thyroid hormone levels, especially in patients treated with levothyroxine (LT4), remains challenging. Moreover, LT4 monotherapy often results in suboptimal thyroid function control, as it may not adequately restore normal free T3 (FT3) levels in many patients (2, 3, 4, 5).

Thyroid function tests, especially serum-free T4 (FT4) and FT3 measurements, are crucial for diagnosing and managing thyroid disorders (6, 7). Various methods exist for measuring thyroid hormones. Mass spectrometry, although highly accurate, is costly and not typically used in clinical settings. Instead, immunoassays are widely employed because of their high sensitivity, specificity, and cost-effectiveness (8). LT4, a synthetic form of T4, is commonly prescribed to restore normal hormone levels. It alleviates symptoms in patients with hypothyroidism. Moreover, thyroid-stimulating hormone (TSH) is the most sensitive marker of thyroid function, and its inverse log-linear relationship with FT4 plays a pivotal role in thyroid homeostasis. Measuring TSH and FT4 simultaneously can be useful in assessing subclinical thyroid dysfunction. Generally, an inappropriate correlation between TSH and FT4 may suggest conditions such as the syndrome of inappropriate secretion of TSH (SITSH). However, patients on LT4 monotherapy often exhibit higher FT4 levels than untreated individuals (9). Notably, this discrepancy has been suggested to result from mechanisms distinct from SITSH and may be specific to LT4 treatment.

In cases of thyroid atrophy or athyreosis, patients may present with normal TSH and FT4 levels but exhibit low FT3 levels during LT4 monotherapy (2, 3, 10). This discrepancy often correlates with low levels of metabolic markers and has been associated with hypothyroid symptoms despite biochemical euthyroidism, which is defined by TSH and FT4 levels alone (2, 11). Increasing the LT4 dosage can normalize FT3 levels by mildly suppressing TSH, resulting in elevated FT4 levels while metabolic markers return to normal (2, 3, 10, 11).

Intra-thyroidal T4-to-T3 conversion and T3 secretion are TSH-dependent, playing a crucial role in thyroid hormone regulation. In addition, various factors, including medication timing, can influence FT4 measurements. Even in patients without thyroid atrophy or athyreosis, factors such as individual variations in thyroid tissue volume, activity of type 2 deiodinase (D2) – an enzyme responsible for converting T4 to T3 – individual metabolic rates, and concurrent medications may affect T4-to-T3 conversion and serum FT3 levels (5, 12, 13, 14). These mechanisms remain incompletely understood and warrant further investigation.

This study aimed to evaluate the effects of varying LT4 dosages on thyroid function, particularly serum FT4 levels. While LT4 therapy is widely used, its dose-dependent impact on FT4 levels remains underexplored. The relationship between elevated FT4 levels in LT4-treated patients and overmedication or physiological requirements for maintaining adequate FT3 levels was investigated, and changes in TSH levels were analyzed.

## Methods

### Patients

This retrospective study analyzed 3020 FT4 measurements from 1,409 patients between July 2021 and March 2024. Data were extracted from the medical record database of Kasahara Clinic following approval from the institutional review board (IRB approval number: 20240401). All patient data were anonymized, and informed consent was obtained using an opt-out method in accordance with the ethical standards of the Declaration of Helsinki. Patients positive for thyrotropin receptor antibodies or treated with antithyroid drugs such as thiamazole, propylthiouracil, and potassium iodide were excluded to eliminate potential confounding factors that could affect thyroid hormone levels independently of LT4 therapy. The cohort included 1,469 measurements from LT4-treated and 1,551 from untreated patients.

### Assessment of overmedication and physiological requirement

Because defining a numerical cutoff to distinguish overmedication from a physiological requirement for elevated FT4 levels is challenging, this distinction was assessed by comparing LT4-treated and untreated groups. Overmedication was primarily defined as FT4 levels above the reference range with markedly suppressed TSH. To further explore whether elevated FT4 levels reflected a physiological requirement, available FT3 measurements were examined.

### Laboratory serum tests

FT4 levels, measured using the Elecsys FT4 III immunoassay (Roche Diagnostics, Switzerland), were categorized by LT4 dose increments of 25 μg. Blood samples were drawn during the day from morning to late afternoon. The reference ranges for thyroid function tests were as follows – FT3: 3.5–6.1 pmol/L; FT4: 12–22 pmol/L; and TSH: 0.61–4.23 mIU/L.

Thyroid volume was estimated by ultrasonography according to the following formula (longitudinal diameter × width × depth × 0.7 cm^3^) for each lobe and the addition of the lobe volumes (15).

### Statistical analysis

Statistical analysis was performed using StatFlex Ver.7 (Artech, Japan), except for the linear mixed-effects model (LMM) and the Jonckheere-Terpstra test, which were conducted using R (version 4.4.3; The R Foundation for Statistical Computing, Austria) (16). Continuous variables were summarized using descriptive statistics, including median and interquartile range (IQR). Normality of continuous variables (FT4, FT3, TSH, and FT3/FT4 ratio) was assessed using the Shapiro–Wilk test. All tested variables showed significant deviation from normality (*P* < 0.001), supporting the use of non-parametric methods for group comparisons and trend analyses.

Comparisons between groups were conducted using the Mann–Whitney U test. For pairwise comparisons among ten LT4 dosage groups (including the untreated group), the Bonferroni correction was applied to adjust for multiple comparisons. This resulted in an adjusted significance threshold of *P* < 0.0011 for 45 pairwise comparisons. Categorical variables were analyzed using the chi-squared test. A *P*-value of less than 0.05 was considered statistically significant unless adjusted for multiple comparisons.

The Jonckheere-Terpstra test (PMCMRplus version 1.9.12) was used to assess trends across increasing LT4 dosages for FT4, FT3/FT4 ratio, TSH, and FT3 levels (17).

Missing values were present for thyroid volume, FT3, TSH, thyroid peroxidase antibody (TPOAb), and thyroglobulin antibody (TgAb). Regression analyses were performed on subsets of cases with complete data for the respective variables. No imputation techniques were applied. Analyses involving FT4, including LMMs, group comparisons, and ROC analysis, were conducted using a complete FT4 dataset.

Because multiple FT4 measurements were obtained from some patients, an LMM was employed using the lme4 package (version 1.1-36) to account for intra-individual correlations (18). For comparisons between LT4-treated and untreated patients, LMM was performed with patient ID as a random intercept and LT4 treatment status as a fixed effect. To analyze the relationship between LT4 dosage and FT4 levels, LMM was performed with LT4 dosage as a fixed effect. Parameter estimation was performed using restricted maximum likelihood.

To identify independent predictors of FT4 levels, a multiple linear regression analysis was performed using the forward stepwise selection method. FT4 concentration (pmol/L) was set as the dependent variable, whereas age, sex, LT4 dosage (μg/day), estimated thyroid volume, TPOAb levels, and TgAb levels were included as independent variables.

Residuals from the LMMs and multiple linear regression analyses were evaluated using the Shapiro–Wilk test and Q–Q plots. Although the residuals exhibited some deviation from normality, particularly due to heavy tails, the large sample size and the robustness of these models to mild normality violations support the validity and interpretability of the model estimates.

## Results

### Baseline characteristics of study participants

The study included 3,020 FT4 measurements from 1,409 patients between July 2021 and March 2024, with a median of two measurements per participant (IQR: 1–3). Of these, 1,469 measurements were from LT4-treated patients, and 1,551 from untreated patients (Table 1). The median age of the entire cohort was 46.0 years (IQR: 35.0–60.0 years). The male-to-female ratio was 1:10.6. In the LT4-treated group, the median age was 46.0 years (IQR: 36.0–63.0 years), whereas in the untreated group, it was 46.0 years (IQR: 34.0–57.0 years). The median LT4 dose in the treated group was 50 μg/day (IQR: 37.5–75.0 μg/day). Thyroid volume data indicated a significantly lower median volume in the LT4-treated group (11.0 mL, IQR: 6.0–21.0 mL) than that in the untreated group (16.0 mL, IQR: 11.0–21.0 mL, *P* < 0.0001).

**Table 1:** Baseline characteristics of study participants. Data are presented as median (IQR).

	LT4 group		Non-LT4 group		*P*-value
	Median (IQR)	*n*	Median (IQR)	*n*	
Age (years)	46.0 (36.0–63.0)	1,469	46.0 (34.0–57.0)	1,551	<0.0001
Sex* (male:female)	87:1,382 (5.9%:94.1%)	1,469	173:1,378 (11.2%:88.8%)	1,551	<0.0001
LT4 dosage (μg/day)	50.0 (37.5–75.0)	1,469	Not applicable		
Estimated thyroid volume (mL)	11.0 (6.0–21.0)	1,242	16.0 (11.0–21.0)	1,414	<0.0001
TSH (mIU/L)	1.99 (0.83–3.48)	1,469	1.76 (0.97–2.94)	1,550	0.059
FT4 (pmol/L)	17.9 (15.6–20.1)	1,469	16.2 (14.5–17.9)	1,551	<0.0001
FT3 (pmol/L)	4.59 (4.12–5.16)	174	4.64 (4.10–5.44)	113	0.32
FT3 (pmol/L)/FT4 (pmol/L) ratio	0.24 (0.20–0.29)	174	0.28 (0.25–0.32)	113	<0.0001

* Sex distribution is shown as the number and percentage of male and female participants in each group.

The *P*-values indicate the statistical significance of the differences between the LT4-treated and non-treated groups for each variable. A *P*-value of less than 0.05 is considered statistically significant, as shown for several variables, including age, gender distribution, estimated thyroid volume, TSH levels, FT4 levels, and the FT3/FT4 ratio. FT3, free triiodothyronine; FT4, free thyroxine; LT4, levothyroxine; TSH, thyroid-stimulating hormone.

For TSH levels, the LT4-treated group had a median of 1.99 mIU/L (IQR: 0.83–3.48 mIU/L), whereas the untreated group had a median of 1.76 mIU/L (IQR: 0.97–2.94 mIU/L), with no statistically significant difference between the two groups. The FT4 levels were significantly different, with the LT4-treated group showing a median FT4 level of 17.9 pmol/L (IQR: 15.6–20.1 pmol/L), compared to 16.2 pmol/L (IQR: 14.5–17.9 pmol/L) in the untreated group (*P* < 0.0001).

The FT3 levels did not differ significantly between the LT4-treated group (median: 4.59 pmol/L, IQR: 4.12–5.16 pmol/L) and the untreated group (median: 4.64 pmol/L, IQR: 4.10–5.44 pmol/L, *P* = 0.32). However, the FT3/FT4 ratio was significantly lower in the LT4-treated group (median: 0.24, IQR: 0.21–0.29) than in the untreated group (median: 0.28, IQR: 0.25–0.32, *P* < 0.0001).

When stratified by LT4 dosage, the basic statistics are as follows. In the subgroup receiving an LT4 dose of less than 25 μg/day (*n* = 12), the median dose was 12.5 μg/day. The category of 25 μg/day or more but less than 50 μg/day (*n* = 369) exhibited a median dose of 25 μg/day. Among those with 50 μg/day or more but less than 75 μg/day (*n* = 560), the median dose was 50 μg/day. In the 75 μg/day or more but less than 100 μg/day bracket (*n* = 336), the median dose was 75 μg/day. The group with 100 μg/day or more but less than 125 μg/day (*n* = 138) had a median dose of 100 μg/day. In the category receiving 125 μg/day or more but less than 150 μg/day (*n* = 40), the median dose was 125 μg/day. For those with 150 μg/day or more but less than 175 μg/day (*n* = 7), the median dose was 150 μg/day. The subgroup with 175 μg/day or more but less than 200 μg/day (*n* = 4) showed a median dose of 175 μg/day, and the highest category, comprising subjects with 200 μg/day or more (*n* = 3), had a median dose of 200 μg/day.

### LMM analysis results

LMM analysis demonstrated a significant association between LT4 dosage and FT4 levels (estimate = 0.042 pmol/L for each 1 μg/day increase in LT4 dosage, 95% confidence interval (CI): 0.038–0.046, *P* < 0.001). The random intercept variance for patient ID was 4.35, and the residual variance was 6.75.

### Distribution of FT4 levels in LT4-treated and untreated groups

**Figure 1:**
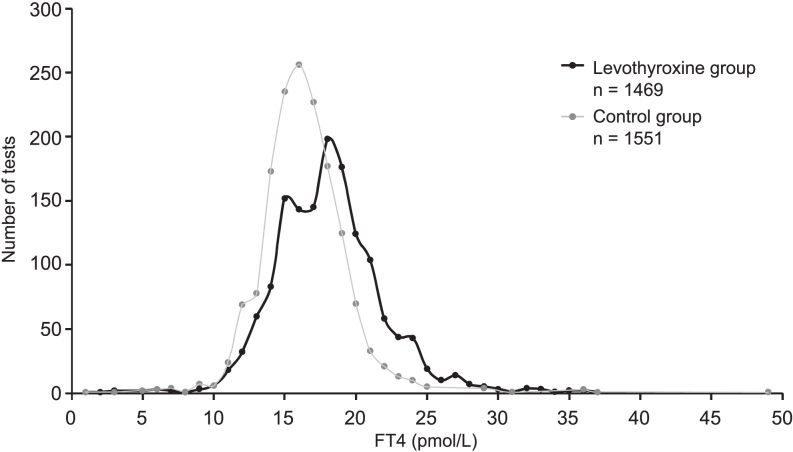
Distribution of FT4 levels (pmol/L) in LT4-treated (black line) and non-treated (gray line) patients. This figure depicts the distribution of free thyroxine (FT4) levels (pmol/L) in patients who are either LT4-treated or non-treated. The x-axis denotes FT4 concentration (pmol/L), and the y-axis represents the number of tests performed. LT4-treated patients are indicated by a black line, whereas non-treated patients are shown with a gray line. The distribution reveals a peak at approximately 18 pmol/L in the LT4-treated group, whereas the non-treated group exhibits a peak of around 16 pmol/L. The FT4 levels for the LT4-treated group predominantly range from 15 to 20 pmol/L, whereas those for the untreated group range from 14 to 18 pmol/L. The histogram suggests that FT4 levels are generally higher in patients receiving LT4 treatment than in those who are not.

The distribution of FT4 levels showed a peak in the LT4-treated group around the 15–20 pmol/L range, whereas the untreated group exhibited a peak around the 14–18 pmol/L range (Fig. 1). LMM analysis demonstrated that FT4 levels were significantly higher in LT4-treated patients than in untreated patients, with a difference of 1.84 pmol/L (95% CI: 1.54–2.14, *P* < 0.0001).

To further explore the relationship between FT4, TSH, and FT3 levels, FT4 was categorized into three groups: < 12 pmol/L, 12–< 22 pmol/L, and ≥ 22 pmol/L. TSH levels decreased progressively with increasing FT4, whereas FT3 levels showed a modest upward trend (Table 2).

**Table 2:** Distribution of TSH and FT3 levels across different FT4 categories. FT4 levels were categorized into three groups: < 12 pmol/L (below the reference range), 12 – <22 pmol/L (within the reference range), and ≥ 22 pmol/L (above the reference range). Median (IQR) values are provided for each parameter. Some data were missing for TSH and FT3 measurements, and the sample sizes (*n*) for each parameter are listed separately.

Category	FT4 (pmol/L)		TSH (mIU/L)		FT3 (pmol/L)	
	*n*	Median (IQR)	*n*	Median (IQR)	*n*	Median (IQR)
FT4 (pmol/L)						
<12	130	11.10 (9.65–11.71)	130	5.525 (2.24–16.96)	13	3.579 (3.084–4.216)
12 – <22	2,653	16.73 (15.06–18.54)	2,652	1.880 (1.040–3.180)	215	4.547 (4.086–5.053)
≥22	237	23.81 (22.65–25.64)	237	0.392 (0.051–1.210)	59	5.130 (4.466–6.163)
Overall	3,020	16.86 (14.93–18.92)	3,019	1.840 (0.908–3.190)	287	4.623 (4.110–5.191)

### Distribution of FT4 and FT3/FT4 levels across different LT4 dosages

**Figure 2:**
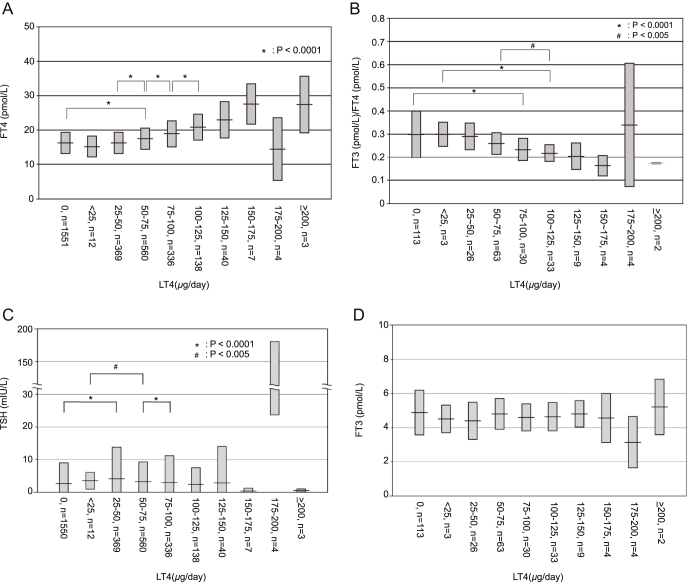
(A) Distribution of FT4 levels across different LT4 dosages. The figure illustrates the distribution of FT4 levels (pmol/L) across various daily dosages of LT4 in patients. The x-axis represents the LT4 dosage categories, whereas the y-axis indicates the FT4 concentration (pmol/L). The LT4 dosage categories are as follows: 0 μg/day (non-treated, *n* = 1,551), 0–25 μg/day (*n* = 12), 25–50 μg/day (*n* = 369), 50–75 μg/day (*n* = 560), 75–100 μg/day (*n* = 336), 100–125 μg/day (*n* = 138), 125–150 μg/day (*n* = 40), 150–175 μg/day (*n* = 7), 175–200 μg/day (*n* = 4), and >200 μg/day (*n* = 3). The histogram shows a higher concentration of FT4 levels in the 50–75 μg/day dosage group, which is significantly higher than that of the non-treated group. *P < 0.0001. In addition, a Jonckheere–Terpstra test revealed a significant positive trend between LT4 dosage and FT4 levels (*P* < 0.001), confirming a dose-dependent increase in FT4 concentrations. (B) Distribution of FT3/FT4 ratios across different LT4 dosages. The figure illustrates the distribution of the FT3/FT4 ratio across various daily dosages of LT4 in patients. The x-axis represents the LT4 dosage categories, whereas the y-axis indicates the FT3/FT4 ratio. The LT4 dosage categories are as follows: 0 μg/day (non-treated, *n* = 113), 0–25 μg/day (*n* = 3), 25–50 μg/day (*n* = 26), 50–75 μg/day (*n* = 63), 75–100 μg/day (*n* = 30), 100–125 μg/day (*n* = 33), 125–150 μg/day (*n* = 9), 150–175 μg/day (*n* = 4), 175–200 μg/day (*n* = 4), and >200 μg/day (*n* = 2). The histogram shows that the FT3/FT4 ratio tends to decrease with increasing LT4 dosage. *P < 0.0001 between the non-treated and the 75–100 μg/day groups, and between the <25 μg/day and the 100–125 μg/day groups; ^#^*P* < 0.005 between the 50–75 μg/day and the 100–125 μg/day groups). In addition, a Jonckheere–Terpstra test revealed a significant negative trend between LT4 dosage and the FT3/FT4 ratio (*P* < 0.0001), confirming a dose-dependent decrease in the FT3/FT4 ratio with increasing LT4 dosage. (C) Distribution of TSH levels across different LT4 dosages. TSH levels across different LT4 dosages. The LT4 dosage categories are as follows: 0 μg/day (non-treated, *n* = 1,550), 0–25 μg/day (*n* = 12), 25–50 μg/day (*n* = 369), 50–75 μg/day (*n* = 560), 75–100 μg/day (*n* = 336), 100–125 μg/day (*n* = 138), 125–150 μg/day (*n* = 40), 150–175 μg/day (*n* = 7), 175–200 μg/day (*n* = 4), and >200 μg/day (*n* = 3). The median TSH levels decreased as LT4 dosage increased. *P < 0.0001 between the non-treated and the 25–50 μg/day groups, and between the 50–75 μg/day and the 75–100 μg/day groups) ^#^*P* < 0.005 between the <25 μg/day and the 50–75 μg/day groups). A Jonckheere–Terpstra test confirmed a significant negative trend (*P* = 0.004), suggesting a dose-dependent suppression of TSH. (D) Distribution of FT3 levels across different LT4 dosages. FT3 levels across different LT4 dosages. The LT4 dosage categories are as follows: 0 μg/day (non-treated, *n* = 113), 0–25 μg/day (*n* = 3), 25–50 μg/day (*n* = 26), 50–75 μg/day (*n* = 63), 75–100 μg/day (*n* = 30), 100–125 μg/day (*n* = 33), 125–150 μg/day (*n* = 9), 150–175 μg/day (*n* = 4), 175–200 μg/day (*n* = 4), and >200 μg/day (*n* = 2). Despite increasing LT4 dosage, FT3 levels remained relatively stable. A Jonckheere–Terpstra test did not show a significant trend (*P* = 0.32), indicating that FT3 levels were not associated with LT4 dosage.

FT4 levels increased with higher LT4 doses, with significant differences observed among dosage groups (*P* < 0.0001). To statistically confirm this trend, a Jonckheere–Terpstra test was performed, revealing a highly significant positive trend between LT4 dosage and FT4 levels (JT = 2,029,623, *P* < 0.0001). These findings provide strong evidence of a dose-dependent increase in FT4 levels with LT4 therapy (Fig. 2A). In addition, the FT3/FT4 ratio tended to decrease with increasing LT4 dosage, showing statistically significant differences compared to the untreated group, indicated by asterisks (*P < 0.0001) and a hash (^#^*P* < 0.005). A Jonckheere–Terpstra test also confirmed a significant negative trend between LT4 dosage and the FT3/FT4 ratio (JT = 9,076, *P* < 0.0001), supporting a dose-dependent decline in the FT3/FT4 ratio with increasing LT4 therapy (Fig. 2B).

TSH levels declined with increasing LT4 dosage (JT = 1,458,745, *P* = 0.004), indicating suppression at higher doses (Fig. 2C). FT3 levels showed a non-significant upward trend with increasing LT4 dosage (JT = 14,929, *P* = 0.32) and were generally within the reference range across all dosage groups (Fig. 2D).

### Association between estimated thyroid volume and FT4 levels

The estimated thyroid volume was significantly smaller in the FT4 ≥ 22 pmol/L group than in the FT4 < 22 pmol/L group (details not shown). However, after excluding patients with estimated thyroid volumes below 5 mL, the FT4 ≥ 22 pmol/L group had a median estimated thyroid volume of 14.0 mL (IQR: 10.0–25.0 mL, *n* = 118), compared to 15.0 mL (IQR: 10.0–21.0 mL, *n* = 2,351) in the FT4 < 22 pmol/L group, with no statistically significant difference (*P* = 0.82). In this subset, LT4 dosage was higher in the FT4 ≥ 22 pmol/L group (median: 50 μg/day, IQR: 0–96 μg/day) compared to the FT4 < 22 pmol/L group (median: 0 μg/day, IQR: 0–50 μg/day), with a statistically significant difference (*P* < 0.0001). FT4 levels exceeding the reference range were primarily associated with LT4 dosage, with minimal influence from thyroid volume.

### Sensitivity and specificity of LT4 dosage in predicting FT4 levels ≥22 pmol/L

**Figure 3:**
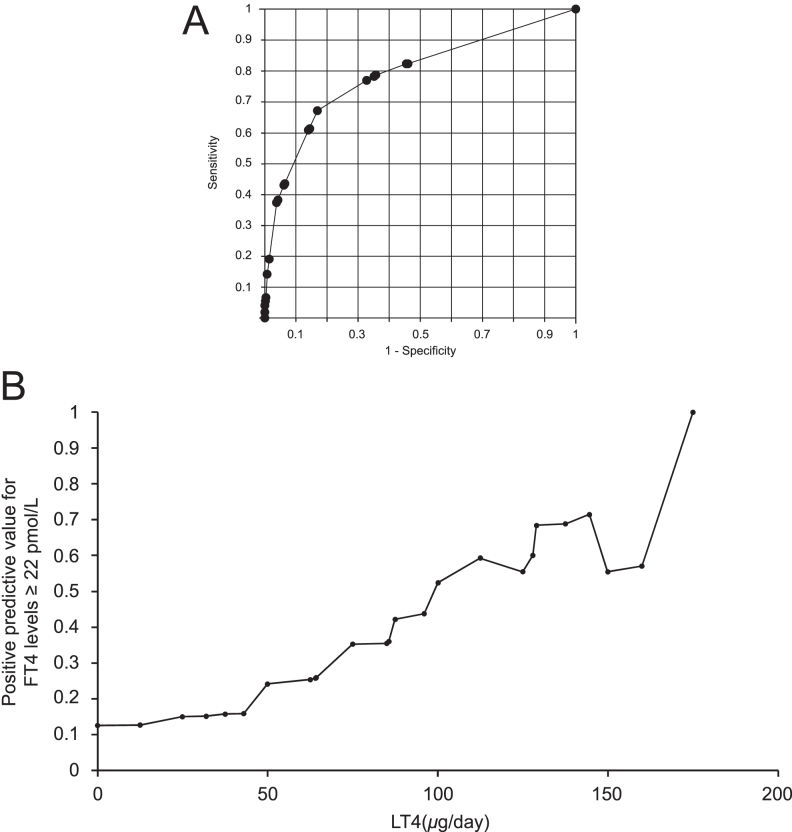
(A) Receiver operating characteristic (ROC) curve for LT4 dosage in predicting FT4 levels ≥22 pmol/L. The ROC curve illustrates the sensitivity and 1-specificity for various LT4 dosage thresholds in predicting elevated FT4 levels (≥22 pmol/L). The optimal cutoff point, where sensitivity equals specificity, is identified at an LT4 dosage of 45.6 μg, with an area under the curve (AUC) of 0.79 (95% CI: 0.75–0.83), indicating moderate predictive accuracy. (B) Positive predictive value (PPV) of LT4 dosage for FT4 levels ≥22 pmol/L. The graph shows the positive predictive value (PPV) for different LT4 dosages in predicting FT4 levels above the upper reference limit of 22 pmol/L. The PPV increases with higher LT4 dosages. This trend suggests a dose-dependent likelihood of elevated FT4 levels in LT4-treated patients.

The sensitivity and specificity of LT4 dosage in predicting FT4 levels of ≥22 pmol/L were analyzed, with the optimal cutoff value – where sensitivity equals specificity – determined to be 45.6 μg (Fig. 3A). At this cutoff, sensitivity and specificity were 0.73. The area under the receiver operating characteristic (ROC) curve for LT4 dosage as a predictor of elevated FT4 levels was 0.79 (95% CI: 0.75–0.83), indicating moderate predictive accuracy. This analysis included 225 participants in the elevated FT4 group and 2,795 in the control group.

The positive predictive values (PPVs) for FT4 levels ≥22 pmol/L at LT4 dosages of 50, 75, and 100 μg were 0.24, 0.35, and 0.52, respectively (Fig. 3B). These results demonstrate an increasing trend in PPV with higher LT4 dosages, indicating a higher likelihood of elevated FT4 levels at these dosage levels.

### Multivariable analysis of FT4 determinants

**Figure 4:**
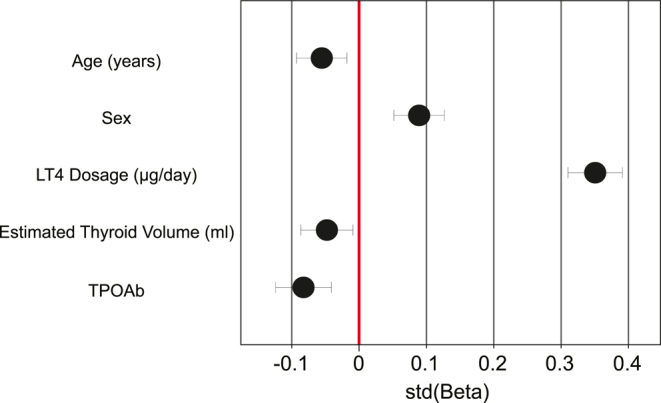
Standardized regression coefficients for predictors of FT4 levels. This figure presents the standardized regression coefficients (stdβ) with 95% confidence intervals from the multiple linear regression analysis predicting free thyroxine (FT4) levels. The x-axis represents standardized β coefficients, whereas the y-axis lists the independent variables included in the model. The red vertical line at 0 indicates no effect. Positive values indicate a direct association with FT4, whereas negative values indicate an inverse relationship. LT4 dosage exhibits the strongest positive association with FT4 levels, followed by sex. Age, estimated thyroid volume, and TPOAb levels show significant negative associations, suggesting their role in modulating FT4 concentrations.

To further investigate the independent factors influencing FT4 levels, a multiple linear regression analysis was performed using the forward stepwise selection method. The model demonstrated a moderate correlation (*R* = 0.37), with an adjusted *R*^2^ value of 0.14, indicating that approximately 14% of FT4 variability could be explained by the included predictors (Fig. 4).

Among the variables analyzed, LT4 dosage showed the strongest positive association with FT4 levels (*β* = 0.039, stdβ = 0.38, *P* < 0.0001), confirming that higher LT4 doses resulted in increased FT4 concentrations. Sex was also a significant factor, with females exhibiting higher FT4 levels than males (*β* = 0.86, stdβ = 0.08, *P* < 0.001).

Age and thyroid volume were negatively associated with FT4, indicating that FT4 levels tended to decline with increasing age (*β* = −0.017, stdβ = −0.08, *P* < 0.0001) and larger thyroid volume (*β* = −0.0098, stdβ = −0.05, *P* = 0.016). Furthermore, higher TPOAb levels were associated with lower FT4 levels (*β* = −0.00020, stdβ = −0.10, *P* < 0.0001), suggesting an autoimmune-related influence on thyroid hormone levels.

TgAb was initially included as a candidate variable but was not significantly associated with FT4 and was excluded from the final model.

## Discussion

This retrospective study analyzed the relationship between LT4 dosage and FT4 levels in LT4-treated patients, focusing on the identification of a clinically relevant threshold for interpreting elevated FT4 levels. These findings indicate that LT4-treated patients frequently exhibit elevated FT4 levels, which appear to be dose-dependent and necessary to maintain adequate FT3 levels (2, 3, 4, 5). Given that some patients had multiple FT4 measurements, a conventional cross-sectional analysis would not have fully captured intra-individual variability. To address this, an LMM was employed, allowing for a more robust estimation of the dose-dependent relationship between LT4 and FT4 levels. This approach strengthens the reliability of these findings and ensures that within-subject variations do not confound the observed association.

The ROC analysis identified a cutoff value of 45.6 μg for predicting FT4 levels exceeding 22 pmol/L; however, its clinical applicability may be limited due to individual variability and other influencing factors. The ROC analysis yielded an AUC of 0.79, indicating moderate predictive accuracy (Fig. 3A). Given this moderate accuracy, the predictive value of this threshold is not absolute, necessitating additional clinical considerations. Therefore, the PPVs for various LT4 dosage levels were analyzed to refine the assessment of FT4 elevations (Fig. 3B). The PPVs for FT4 levels above the upper reference limit increased with higher LT4 dosages, reaching 0.24, 0.35, and 0.52 for 50, 75, and 100 μg, respectively. Approximately one-third of patients receiving 75 μg of LT4 had FT4 levels exceeding the reference range, suggesting that this dosage level could represent a more practical threshold for interpreting elevated FT4 values. Therefore, using 75 μg as a clinical reference point for assessing FT4 levels may offer a balanced approach, allowing clinicians to distinguish physiological elevations from potential overmedication, and avoid unnecessary dose reductions that could compromise FT3 levels. This approach aligns with observed treatment responses, where maintaining FT3 levels often necessitates a higher FT4 concentration in LT4 monotherapy.

Comparing these findings to previous research, we see a distinction in the role of thyroid volume and LT4 dosage. Recent studies have highlighted various factors that can influence thyroid hormone levels in patients undergoing LT4 therapy (19). Prior studies have reported that smaller thyroid volumes are associated with higher FT4 levels, whereas larger thyroid volumes correspond with elevated FT3 owing to enhanced T4-to-T3 conversion (5). This study adds new insights by demonstrating that, after excluding patients with thyroid volumes below 5 mL, FT4 elevations were primarily associated with LT4 dosage rather than thyroid volume. This finding was further supported by multivariable analysis, which showed that LT4 dosage had a stronger association with FT4 levels than thyroid volume. It is clinically relevant, as it suggests that thyroid volume may have a limited impact on FT4 levels in most LT4-treated patients, reinforcing LT4 dosage as the critical factor.

These results indicate that elevated FT4 levels in LT4-treated patients should not be immediately interpreted as overmedication. Some patients on LT4 monotherapy exhibit FT4 levels that exceed the reference range (20, 21). Given the critical roles of T4 and T3 in the body, it is essential to closely monitor their levels to ensure effective treatment. Recent studies highlight the complexities of thyroid hormone replacement therapy, particularly in achieving optimal FT3 levels with LT4 monotherapy (2, 3, 4, 5). These findings are consistent with the work of Gereben *et al.*, who identified a ubiquitin-proteasome-mediated mechanism responsible for the selective proteolysis of D2, thereby modulating the T4-to-T3 conversion in response to elevated T4 levels (12). This proteolytic regulation helps prevent excessive T3 production in the presence of high T4 levels, potentially explaining why FT3 levels remain stable while FT4 levels rise in LT4-treated patients (12, 13, 14). This regulatory mechanism supports the idea that elevated FT4 levels observed in LT4 monotherapy reflect a physiological adaptation necessary to maintain adequate FT3 levels in peripheral tissues. In addition, the mild but significant suppression of TSH with increasing LT4 dosage reduces thyroidal T3 synthesis, whereas FT4-induced ubiquitination of D2 limits T4-to-T3 conversion, collectively contributing to a lower FT3/FT4 ratio. These results indicate that even when TSH remains within the reference range, LT4 dosage significantly influences FT4 levels. Consequently, maintaining FT3 levels within the reference range in LT4-treated patients may necessitate a slightly elevated FT4 concentration.

Consequently, evaluating FT4 and FT3 levels, along with the FT3/FT4 ratio, may offer a more comprehensive assessment of thyroid function in LT4-treated patients.

The clinical implications of these findings are significant, as misinterpretation of elevated FT4 levels could lead to inappropriate dose reductions, resulting in suboptimal management of hypothyroidism. Given the short half-life of FT3, FT4 is more commonly measured; however, reducing LT4 dosage based solely on FT4 elevations risks inadequate treatment (22). The findings suggest that clinicians should interpret FT4 levels within the broader context of thyroid hormone physiology and avoid unnecessary dose adjustments that might lead to reduced FT3 availability. This approach is consistent with the broader objectives of hypothyroidism management, which aim to stabilize FT4 and FT3 levels to meet individual physiological demands.

Future studies should explore the impact of genetic variations and deiodinase activity on thyroid hormone metabolism, as these factors may further refine LT4 dosing strategies in individual patients. In addition, combining LT4 with slow-release T3 formulations may offer an alternative strategy for those unable to maintain adequate FT3 levels on LT4 monotherapy alone. The reference range for FT4 is based on healthy individuals without thyroid disease, and it may not always be applicable to patients on LT4 therapy. Elevated FT4 levels in these patients may reflect physiological needs rather than inappropriate TSH suppression or overmedication. Clinicians should, therefore, evaluate FT4 levels within the context of treatment goals rather than relying solely on reference ranges established for the general population.

Although this study provides valuable insights, there are limitations to consider. While the overall sample size was large, the number of patients receiving higher LT4 dosages (e.g. ≥150 μg/day) was relatively small, and FT3 measurements were available for only a subset of patients. These factors may have limited the ability to fully evaluate FT3 dynamics across all dosage levels. Additional limitations include variability in assay methods for thyroid hormone measurement, and potential differences in clinical practice and patient demographics. Owing to the retrospective nature of this study, specific tests to exclude the possibility of non-specific reactions, such as the polyethylene glycol precipitation recovery test, could not be performed. As a result, non-specific interactions affecting FT4 levels may not be fully excluded from the analysis. Although appropriate statistical models were employed, including LMMs and multiple linear regression, some deviation from normality in residuals was observed. Further research is needed to confirm these findings across diverse patient populations and to explore longitudinal outcomes associated with different LT4 dosing strategies (23, 24). Understanding the individual factors that influence FT4 and FT3 levels in LT4-treated patients, including genetic variations and deiodinase activity, could ultimately contribute to a more personalized approach to hypothyroidism management.

## Conclusion

FT4 levels are higher in LT4-treated patients and increase with higher doses. Elevated FT4 levels in these patients reflect physiological needs rather than overmedication. Clinicians should consider these findings for optimal management of hypothyroidism.

## Declaration of interest

The author declares no competing financial interests or personal relationships that could have influenced the work reported in this paper.

## Funding

This research did not receive any specific grant from any funding agency in the public, commercial, or not-for-profit sector.

## Author contribution statement

TK conceptualized the study, conducted data processing, and drafted the manuscript.

## Data access statement

The data used in this study were obtained from patient records at Kasahara Clinic and contain sensitive patient information. Owing to privacy and confidentiality agreements, the raw data are not publicly available. Aggregated data are available from the author upon reasonable request.
